# The Enhancement of Regulatory T Cell Maturation and Th1/Th2 Balance through FOXP3 Expression by *Lactobacillus paracasei* in an Ovalbumin-Induced Allergic Skin Animal Model

**DOI:** 10.3390/cimb46100636

**Published:** 2024-09-24

**Authors:** Chin-Feng Liu, Wen-Yu Chao, Tsung-Wei Shih, Chun-Lin Lee, Tzu-Ming Pan

**Affiliations:** 1 Continuing Education Program of Food Biotechnology Applications, National Taitung University, Taitung 950017, Taiwan; cfliu@nttu.edu.tw; 2 SunWay Biotech Co., Taipei 114063, Taiwan; wendy.chao@sunway.cc (W.-Y.C.); tw.shih@sunway.cc (T.-W.S.); 3 Department of Life Science, National Taitung University, Taitung 950309, Taiwan; 4 Department of Biochemical Science and Technology, National Taiwan University, Taipei 106216, Taiwan

**Keywords:** atopic dermatitis (AD), NTU 101, Th1/Th2 balance, neutrophil maturation

## Abstract

Chronic allergic skin conditions, including atopic dermatitis (AD), are characterized by pruritus, erythema, xerosis, desquamation, and inflammation, significantly impacting quality of life. Long-term steroid use, while common in treatment, carries the risk of adverse effects. Previous studies have demonstrated the potential of *Lactobacillus paracasei* subsp. *paracasei* NTU 101 (NTU 101) in alleviating AD symptoms from a preventive perspective. This study, however, focuses on exploring NTU 101’s therapeutic potential by investigating its effects on regulatory T cell (Treg) maturation and Th1/Th2 balance. The results revealed that NTU 101 administration effectively reduced serum IgE levels and inflammatory cell infiltration in the skin, leading to a significant improvement in both epidermal and dermal thickness in the AD model. Additionally, NTU 101 modulated the immune response by increasing the proportion of CD4+/IL-4+ (Th2) cells in the spleen and concurrently enhancing FOXP3 expression in CD4+/CD25+ cells, which is critical for Treg cell development. This immune modulation was further associated with a rebalancing of the Th1/Th2 ratio, achieved by increasing the proportion of CD4+/IFN-γ+ (Th1) cells. Moreover, NTU 101 influenced the proportion of CD4+IL-17+ (Th17) cells, thereby supporting neutrophil maturation and promoting allergen clearance, ultimately mitigating AD symptoms. These findings underscore the potential of NTU 101 not only in managing AD symptoms but also in modulating key immune pathways involved in the pathogenesis of the disease, offering a promising alternative or adjunct to conventional steroid therapies.

## 1. Introduction

Atopic dermatitis (AD) is a chronic and recurrent skin condition commonly observed in both children and adults. The inflammatory responses in AD include symptoms such as redness, itching, and swelling, significantly impacting the quality of life and leading to higher rates of anxiety, depression, and reduced self-esteem [1]. Environmental factors such as air pollution, allergens, and climate conditions exacerbate AD symptoms, complicating its management. Genetic predisposition also plays a crucial role in the development and severity of AD, with certain mutations in the filaggrin gene being linked to skin barrier dysfunction. Traditional treatment methods for AD involve a combination of topical steroids, antihistamines, emollients, and systemic medications. Traditional treatments for AD involve topical steroids, antihistamines, emollients, and systemic medications to reduce inflammation, alleviate itching, and repair the skin barrier. In severe cases, immunosuppressive drugs and biologics may be prescribed, but prolonged use carries risks such as skin thinning, hormonal imbalances, and increased infection risk, with limited therapeutic efficacy for some patients [2]. Recent therapeutic options include biological therapies and JAK inhibitors, which have shown promising results in managing AD by targeting specific pathways involved in the inflammatory process [3].

Probiotics are live microorganisms that, when administered in adequate amounts, confer health benefits to the host by regulating the balance of microorganisms in the gastrointestinal tract. To date, probiotic research has mainly focused on their effects on gut health and overall health, including their impact on gut microbiota balance, digestive function, immune system, and mental health [4]. The potential applications of probiotics in preventing and treating intestinal diseases, inflammatory diseases, allergic reactions, cardiovascular diseases, and other conditions have also been explored [5]. Probiotics have been found to modulate the immune response by enhancing the activity of certain immune cells, reducing inflammation, and promoting the production of anti-inflammatory cytokines. Additionally, they may help maintain the integrity of the gut barrier, preventing the translocation of harmful pathogens and toxins into the bloodstream. In recent years, the concept of probiotics influencing skin health through the gut–skin axis has gained attention, suggesting that a healthy gut microbiome can contribute to healthier skin [6]. Probiotic treatments have also shown potential benefits for other skin disorders, such as psoriasis, eczema, and acne, indicating a broader application of these beneficial microbes in dermatology [7].

Studies show that probiotic supplementation can reduce AD incidence by modulating immune responses [8]. Clinical trials have indicated that early supplementation of specific probiotics, such as *Lactobacillus rhamnosus*, in pregnant women and newborns can decrease the risk of infants developing AD. This is attributed to probiotics’ ability to adjust the infant’s gut microbiota and strengthen the immune system, thereby enhancing tolerance to environmental allergens [8]. Long-term observational studies indicate that consistent probiotic intake during early childhood may offer sustained protection against AD and other allergic conditions, emphasizing the importance of early and continuous supplementation. Therapeutically, probiotics have shown potential in improving skin barrier function and reducing inflammation. Clinical studies indicate that oral or topical probiotics, particularly from Bifidobacterium and Lactobacillus strains, can significantly improve AD symptoms by reducing itching and inflammation [9]. This effect may be due to probiotics regulating the balance of Th1/Th2 immune responses and increasing the production of anti-inflammatory cytokines [4]. Additionally, emerging research suggests that combining probiotics with prebiotics or conventional AD treatments may enhance their efficacy, offering a comprehensive approach to managing AD symptoms and improving quality of life [10].

Furthermore, the mechanisms by which probiotics exert their effects on AD are being actively investigated. Probiotics may reduce allergen penetration and allergic reactions by improving intestinal barrier function and can directly act on the skin by producing specific metabolites, such as short-chain fatty acids, which enhance skin barrier integrity and suppress inflammatory responses. These metabolites have systemic anti-inflammatory effects, aiding in the management of inflammatory skin diseases like AD. Additionally, probiotics may modulate the immune system by promoting the development and function of regulatory T cells (Tregs), which play a crucial role in maintaining immune homeostasis and preventing excessive inflammatory responses. These findings provide a scientific basis for the use of probiotics in managing AD [11]. The gut–skin axis is a concept that highlights the bidirectional relationship between the gastrointestinal system and skin health [12]. Probiotics can influence this axis by maintaining or restoring a healthy gut microbiome, thereby improving skin conditions. Imbalances in gut microbiota, often caused by poor diet, stress, and antibiotics, are linked to various inflammatory and autoimmune diseases, including AD. By restoring the balance of beneficial bacteria, probiotics can help manage and prevent these conditions [9]. This holistic approach underscores the importance of considering both gut and skin health in the treatment and prevention of AD, and it suggests that probiotic supplementation could be a valuable adjunctive therapy in comprehensive AD management strategies. 

Integrating probiotics into the management of AD through dietary approaches offers a promising adjunct to traditional treatments. By modulating the gut microbiota and enhancing immune responses, probiotics provide a potential strategy to reduce the incidence and severity of AD. Specific strains of probiotics have shown unique benefits in the context of AD. For example, Bifidobacterium breve and *Lactobacillus paracasei* have demonstrated potential in clinical trials to reduce the severity of eczema and improve skin hydration [13]. These strains work by increasing the production of mucins in the gut, which helps maintain intestinal barrier integrity, and by producing antimicrobial peptides that directly inhibit pathogenic bacteria [14].

In prior research, our team utilized a preventive animal model to explore the efficacy of NTU 101 in averting AD. The results showed that NTU 101 led to a notable reduction in AD symptoms. It is hypothesized that the preventive mechanism of NTU 101 includes the upregulation of Forkhead box protein P3 (FOXP3) and the promotion of regulatory T cell (Treg cell) maturation, which in turn alleviates the allergic reactions associated with AD [15]. Building upon these findings, our ongoing studies are now shifting focus to the therapeutic model, aiming to further investigate how NTU 101 can be applied to treat existing AD. The therapeutic approach is critical because it examines NTU 101’s potential not only to prevent but to actively reverse disease progression, mitigate symptoms in already affected individuals, and explore its mechanisms in modulating immune responses, enhancing skin barrier function, and reducing inflammation. Understanding these effects is essential for determining how NTU 101 may offer comprehensive benefits in AD treatment. These findings are expected to shed new light on the future application of NTU 101 in the treatment of AD and expand its role beyond prevention.

## 2. Materials and Methods

### 2.1. Sample Preparation

*Lactobacillus paracasei* subsp. paracasei NTU 101 (NTU 101) in powder form, with a concentration of 1 × 10^11^ CFU/g, was sourced from SunWay Biotech Co., Ltd., Taipei, Taiwan. Another strain, *Lactobacillus rhamnosus* ATCC 53103, was obtained from the Bioresource Collection and Research Center in Hsinchu, Taiwan, and prepared as a lyophilized powder with a stable viable count of 1 × 10^11^ CFU/g. Prednisolone, a synthetic corticosteroid widely used for treating severe allergies, was procured from Sigma-Aldrich in St. Louis, MO, USA. Ovalbumin (OVA), used for studying antigen-specific immune responses in mice as a T cell-dependent antigen, was administered in two main ways: (1) For systemic sensitization, OVA (20 µg) was combined with an adjuvant (2 mg of Al(OH)_3_ in 0.2 mL of sterile 0.9% saline) and administered via intraperitoneal injection. The saline solution was autoclaved and settled at room temperature for at least 30 min before use; (2) For local skin irritation and sensitization, 100 µg of OVA was dissolved in 100 µL of 0.9% saline and applied directly to the skin [16,17].

### 2.2. Animal Grouping and Experiment Schedule

Five-week-old Balb/c mice were acquired from BioLasco Co. (Taipei, Taiwan). Upon arrival, the mice were randomly assigned to groups and housed in stainless steel cages in a controlled environment, maintained at a relative humidity of 50 ± 10% and a temperature of 23 ± 2 °C, with a 12 h light–dark cycle. Each cage was externally labeled with an animal identification card, as required by the “Animal and Raw Material Management Procedure” (ATRI-ATL-QP-008). These cards displayed essential information, including the IACUC number (No. 106038), strain, sex, group allocation, individual animal numbers, birth dates, testing cycles, and handling protocols. Prior to the commencement of substance testing, the mean body weight of the mice was recorded at 15.52 ± 0.04 g, with intergroup variations kept below 20%.

**Figure 1:**
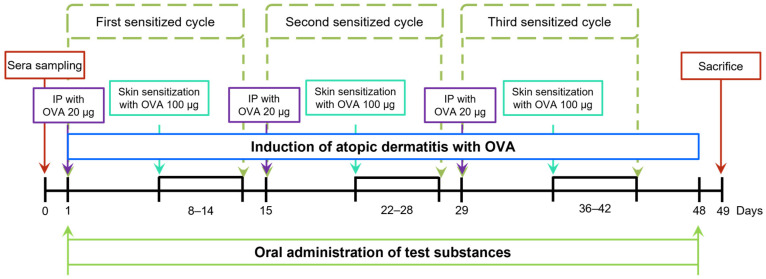
Experimental schedule in this study.

Forty mice were randomly divided into five groups for the study. The NOR group, serving as the control, received saline and was maintained on a standard diet. The OVA group underwent systemic sensitization and local skin irritation with ovalbumin (OVA) while continuing a normal diet. The CM group was similarly sensitized and irritated with OVA but also received concurrent prednisolone treatment with no changes to their diet. The NTU 101 group underwent OVA sensitization and irritation, with NTU 101 administered at a dose of 1 × 10^11^ CFU/day while on a normal diet. The LGG group followed the same OVA sensitization and irritation procedures but was pre-fed *L. rhamnosus* GG ATCC 53103 at a dose of 1 × 10^11^ CFU/day, maintaining a normal diet throughout the experiment. Food consumption was monitored weekly by recording the amount of food provided and the remaining amount. During the OVA-induction phase, each mouse received an intraperitoneal injection of OVA (20 μg/200 μL) over a one-week period to initiate systemic sensitization. Subsequently, the skin was topically stimulated with OVA at a concentration of 100 μg/100 μL per mouse, applied daily for 7 days. As depicted in Figure 1, this cycle was repeated three times. To simulate human administration, all test substances were administered intragastrically for 49 consecutive days.

### 2.3. AD Score, SCORE

In this study, skin conditions were evaluated after stimulating the skin with OVA, with observations, scoring, and photographing conducted every 3 to 4 days. The scoring of skin conditions followed these criteria: 0 points for no symptoms, 1 point for scratching, 2 points for redness, 3 points for skin damage or flaking, 4 points for mucus or body fluid production, and 5 points for the presence of mucus or body fluids with pus or bleeding.

### 2.4. Blood Collection and Analysis

Blood samples were first collected from the orbital plexus into tubes without anticoagulant. After the animals were sacrificed, additional blood was drawn directly from the heart into tubes, either with or without anticoagulant. Blood collected without anticoagulant was centrifuged at 3500 rpm for 10 min at 4 °C, and the resulting supernatant was stored at −40 °C for later use. For hematological analysis, whole blood was treated with EDTA and analyzed using a ProCyte Dx hematology analyzer (IDEXX Laboratories, Westbrook, MA, USA) for a five-part differential white blood cell count, which included neutrophils, lymphocytes, monocytes, eosinophils, and basophils, following protocol ATRI-ATL-ISOP-027.

### 2.5. Enzyme-Linked Immunosorbent Assay (ELISA)

The levels of total IgE in the serum were measured before and after the experiment, while the levels of OVA-specific IgE were measured post-experiment. Commercial kits are used to determine the concentrations of total IgE (Cat. No. BTLE99–115, Bethyl, Montgomery, TX, USA) and OVA-specific IgE (Cat. No. BRAMCA2475KZZ, Bio-rad, Hercules, CA, USA). All measurements were performed according to the protocols provided in the respective kit manuals.

### 2.6. Cell Clustering and Cytokine Detection

Splenocytes were prepared and analyzed using flow cytometry to assess the expression of various immune cell markers, including CD4+/IL-4+, CD4+/IL-17+, CD4+/IFN-γ+, and CD4+/CD25+/FOXP3+. To isolate the splenocytes, the spleen was first minced with sterile scissors and then dissociated through a 70 µm cell strainer using the plunger of a 10 mL syringe. The cells were suspended in 15 mL of RPMI 1640 medium at 4 °C, centrifuged at 1000 rpm for 10 min, and washed twice with PBS. The cell pellets were treated with ammonium–chloride–potassium (ACK) lysis buffer (Becton Dickinson) at room temperature and gently mixed for 10 min to lyse red blood cells. After lysis, the cells were centrifuged again and washed with 1× PBS at 4 °C to obtain a pure splenocyte suspension. For staining, 1 mL of freshly isolated splenocytes was incubated with 10 µL of propidium iodide (PI) (50 μg/mL) for 10 min in the dark to prepare for flow cytometric analysis. The presence and proportion of the specific CD markers were detected using a flow cytometer and analyzed using WinMDI 2.9 software (Scripps Research Institute, La Jolla, CA, USA).

### 2.7. Detection of Immune Cell Populations Using Monoclonal Antibodies and Flow Cytometry

To analyze immune cell populations using flow cytometry, red blood cells were removed from spleen cell suspensions using ACK lysis buffer (Becton Dickinson, San Diego, CA, USA). The erythrocyte-depleted spleen cells were resuspended in FACS buffer (BD) and then fixed and permeabilized using the BD Cytofix/Cytoperm™ Kit (Cat. No. 554714, BD). Cell staining was performed using a panel of monoclonal antibodies: FITC-labeled anti-mouse CD4 (clone: RM4-5, eBioscience, San Diego, CA, USA); PE-labeled rat anti-mouse CD25 (clone: PC61.5, eBioscience); PerCP-Cy™ 5.5-labeled rat anti-mouse IFN-γ (clone: XMG1.2, BD, NJ, USA); PerCP-Cy™-labeled rat anti-mouse IL-4 (clone: 11B11, BioLegend, San Diego, CA, USA); PerCP-Cy™ 5.5-labeled rat anti-mouse FOXP3 (clone: FJK-16s, Invitrogen, Carlsbad, CA, USA); and PerCP-Cy™ 5.5-labeled rat anti-mouse/rat IL-17A (clone: eBio17B7, eBioscience). The flow cytometer was used to analyze fluorescence intensity, identifying CD4+ cells as Th cells, CD4+IFN-γ+ as Th1 cells, CD4+/IL-4+ as Th2 cells, CD4+/IL-17+ as Th17 cells, and CD4+CD25+FOXP3+ as Treg cells. Data analysis was performed using WinMDI software (version 2.9).

### 2.8. Histopathological Analysis

Skin tissue from the stimulated area was collected and fixed in neutral 10% formalin solution prior to histological sectioning and pathological analysis. The tissue was cross-sectioned and placed in embedding cassettes. It was then dehydrated, infiltrated with paraffin, and embedded into paraffin blocks. Sections 4 µm thick were cut using a paraffin microtome (Leica RM 2145, Nussloch, Germany). These sections were stained with Hematoxylin & Eosin (H&E) and assessed using immunohistochemistry (IHC) to measure thymic stromal lymphopoietin (TSLP) antigen levels (Cat. No. PA5–20320, Thermo, Waltham, MA, USA). The extent of AD induced by OVA stimulation was evaluated under a light microscope [18].

### 2.9. Statistical Analysis

All data calculations and presentations were performed using Microsoft Excel from the Office commercial software suite, calculating mean values and standard deviations (SD), displayed as “Mean ± SD”. Statistical analyses were performed using SPSS version 12.0, utilizing One-Way ANOVA with Dunnett’s *t*-test or Scheffe’s post hoc test for data analysis. The expression of TSLP antigen was analyzed using the Mann–Whitney U-test. Comparisons were made between the NTU 101, LGG, CM, NOR, and OVA groups, with a *p*-value of less than 0.05 considered statistically significant. Asterisks denote significance levels: * *p* < 0.05, ** *p* < 0.01, *** *p* < 0.001. The choice of these statistical methods was based on the nature and distribution of the data, with One-Way ANOVA and post hoc tests appropriate for comparing means across multiple groups and the Mann–Whitney U-test suitable for non-parametric data comparison. These methods were selected to ensure the accuracy and reliability of the data analysis.

## 3. Results

### 3.1. Observation of Food Intake and Body Weight Changes during the Experimental Period

**Figure 2:**
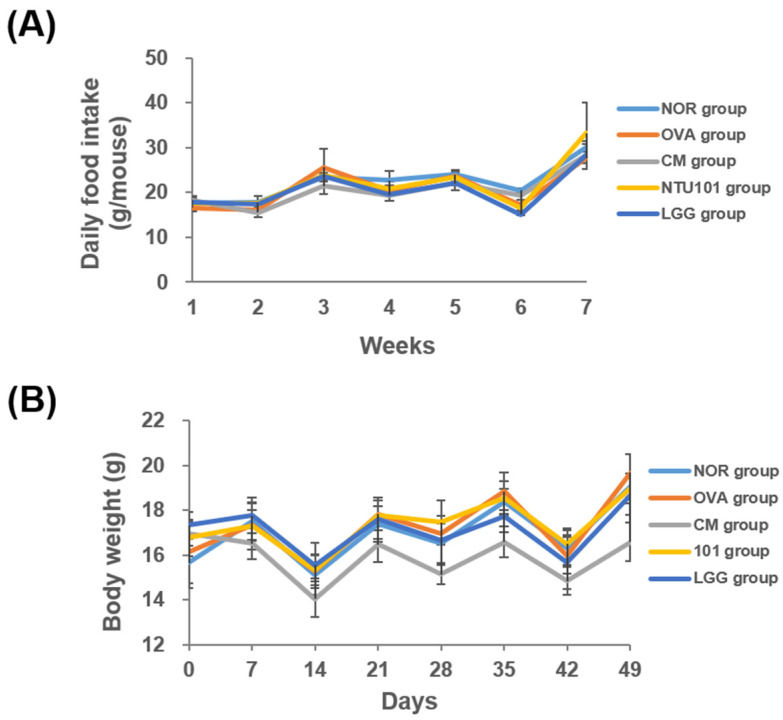
Food intake and body weight during the 7-week experiment. (**A**) Weekly food intake (g/mouse). (**B**) Body weight (g/mouse). Each value was expressed as mean ± SD (*n* = 8). The NOR group (control) received saline and a standard diet. The OVA group underwent sensitization and skin irritation with ovalbumin (OVA) on a normal diet. The CM group had the same OVA treatment plus prednisolone. The NTU 101 group received OVA treatment and NTU 101 at 1 × 10^11^ CFU/day. The LGG group underwent OVA treatment and received *L*. *rhamnosus* GG ATCC 53103 at 1 × 10^11^ CFU/day.

During the experimental period, food intake decreased across all groups during the skin-stimulation process used to induce the AD animal models, while it increased during systemic sensitization. This fluctuation in food intake was attributed to the need for bandaging to secure the stimulation process, which may have affected the animals’ appetite due to discomfort. Despite this, food intake was similar across all groups throughout the experiment, and no statistically significant differences were observed (Figure 2A).

In terms of body weight, the average weekly weight of the animals in each group is shown in Figure 2B. During the skin stimulation induction period, the average weight of animals in all groups decreased, likely due to the discomfort caused by continuous bandaging for 7 days to ensure consistent contact between OVA and the skin. This discomfort led to reduced food intake and subsequent weight loss. However, after the bandages were removed, the animals’ weight increased. Throughout the experiment, there was no statistical difference in weight between the OVA and NOR groups. The weight of animals in all groups was similar to the NOR group, with two exceptions: the LGG group had significantly lower weight compared to the OVA group on Days 35 and 49 (*p* < 0.05), and the CM group showed significantly lower weight than the OVA group on Day 14 after receiving prednisolone (*p* < 0.05), as well as on Days 21, 28, 35, 42, and 49 (*p* < 0.05, *p* < 0.01, *p* < 0.001).

### 3.2. Probiotics Improve Skin Conditions in Individuals with AD

**Figure 3:**
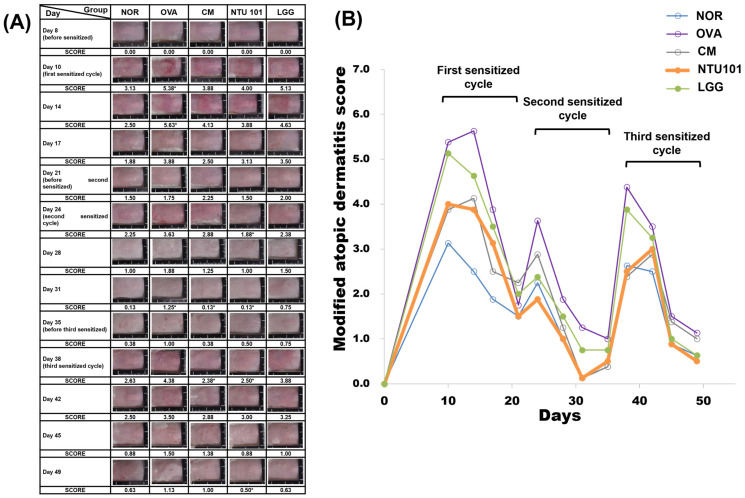
(**A**) Representative images of sensitized skin sites in EC-sensitized mice. (**B**) Clinical skin severity scores. Each value was expressed as mean ± SD (*n* = 8). Statistical comparison of differences between each group: * denotes *p*-value less than 0.05 (*p* < 0.05). The NOR group (control) received saline and a standard diet. The OVA group underwent sensitization and skin irritation with ovalbumin (OVA) on a normal diet. The CM group had the same OVA treatment plus prednisolone. The NTU 101 group received OVA treatment and NTU 101 at 1 × 10^11^ CFU/day. The LGG group underwent OVA treatment and received *L. rhamnosus* GG ATCC 53103 at 1 × 10^11^ CFU/day.

The summarized results of skin condition observations for each group induced with AD animal models using OVA are shown in Figure 3. Skin conditions were recorded every 3 to 4 days during the induction period, which involved a total of three rounds of skin stimulation. During the first round (Days 10 and 14), the average skin score at the stimulation site for the OVA group was 5.38 and 4.63, significantly higher than the average scores of 3.13 and 2.50 for the NOR group (*p* < 0.05; *p* < 0.01). The CM group had average scores of 3.88 and 4.13, the NTU 101 group scored 4.00 and 3.88, and the LGG group scored 5.13 and 4.63. A comparison with the OVA group showed that the NTU 101, LGG, and CM groups all had lower average skin scores at the stimulation site, with the NTU 101 group also scoring lower than the LGG group and similar to the CM group, although these differences did not reach statistical significance. When the animals were not in contact with the sensitizing substance (OVA), their skin conditions gradually returned to normal. On Days 17 and 21, the average skin scores at the stimulation site were 1.88 and 1.50 for the NOR group, 3.88 and 1.75 for the OVA group, 2.50 and 2.25 for the CM group, 3.13 and 1.50 for the NTU 101 group, and 3.50 and 2.00 for the LGG group. It was observed that the NTU 101 group had lower average skin scores at the stimulation site compared to the LGG group, and the scores approached those of the CM and NOR groups.

During the second round of skin stimulation with OVA (Days 24 and 28), the severity of skin inflammation in the OVA group was alleviated compared to the first round, with a shorter recovery time. The average skin scores at the stimulation site for the CM group were 2.88 and 1.25, 1.88 and 1.00 for the NTU 101 group, and 2.38 and 1.50 for the LGG group. The average scores for these three groups were lower than those of the OVA group (3.63 and 1.88), with the NTU 101 group showing the lowest scores, closest to the NOR group (2.25 and 1.00). During the non-stimulation period (Day 31), the average skin score for the OVA group was 1.25, higher than that of the NOR group (0.13, *p* < 0.05), while the CM and NTU 101 groups both had scores of 0.13, significantly lower than the OVA group (*p* < 0.05)

During the third round of skin stimulation (Days 38 and 42), the average skin scores at the stimulation site were 2.63 and 2.50 for the NOR group, 4.38 and 3.50 for the OVA group, 2.38 and 2.88 for the CM group, 2.50 and 3.00 for the NTU 101 group, and 3.88 and 3.25 for the LGG group. The OVA group had higher average skin scores at the stimulation site than the NOR group, while the NTU 101 and CM groups had lower scores compared to the OVA and LGG groups, approaching those of the NOR group. When the animals were not in contact with the sensitizing substance (Days 45 and 49), the average skin scores at the stimulation site were 0.88 and 0.63 for the NOR group, 1.50 and 1.13 for the OVA group, 1.38 and 1.00 for the CM group, 0.88 and 0.50 for the NTU 101 group, and 1.00 and 0.63 for the LGG group. It was observed that the skin condition in the NTU 101 group recovered the fastest among all treatment groups and approached that of the NOR group, although these differences did not reach statistical significance.

### 3.3. Therapeutic Effect of Probiotics on AD through Histopathological Analysis

**Figure 4:**
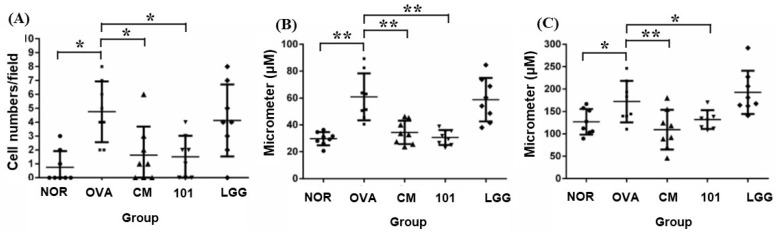
Histological changes in inflammatory cell infiltration (**A**), epidermal thickness (**B**), and dermal thickness (**C**) in each group. H&E staining at 100–200× magnification and 500-micrometer (μm) ruler length. Each value was expressed as the mean ± SD (*n* = 8). Statistical comparison of differences between each group: * denotes *p*-value less than 0.05 (*p* < 0.05); ** denotes *p*-value less than 0.01 (*p* < 0.01). The NOR group (control) received saline and a standard diet. The OVA group underwent sensitization and skin irritation with ovalbumin (OVA) on a normal diet. The CM group had the same OVA treatment plus prednisolone. The NTU 101 group received OVA treatment and NTU 101 at 1 × 10^11^ CFU/day. The LGG group underwent OVA treatment and received *L. rhamnosus* GG ATCC 53103 at 1 × 10^11^ CFU/day.

After sacrificing the animals, skin samples from the stimulated areas were subjected to both H&E staining and immunohistochemical staining with an anti-TSLP antibody. Four indicators were assessed: inflammatory cell infiltration (Figure 4A), thickness of the epidermal cell layer (acanthosis) (Figure 4B), thickness of the dermal layer (Figure 4C), and expression of TSLP antigen (Figure 4). Regarding inflammatory cell infiltration, previous studies suggest that eosinophils predominate in the lesion areas, accompanied by a small number of monocytes. At 100× magnification, the average number of inflammatory cells was 0.7 ± 1.2 in the NOR group, 4.7 ± 2.2 in the OVA group, 1.6 ± 2.1 in the CM group, 1.5 ± 1.5 in the NTU 101 group, and 4.1 ± 2.6 in the LGG group. The number of inflammatory cells in the OVA group was significantly higher than in the NOR group (*p* < 0.05). In contrast, the NTU 101 and CM groups showed significantly lower inflammatory cell infiltration compared to the OVA group (*p* < 0.05) (Figure 4A). Regarding the thickness of the epidermal cell layer, measurements at 200× magnification showed that the average thickness in the lesion area was 29.7 ± 5.0 µm in the NOR group, 60.8 ± 17.5 µm in the OVA group, 34.4 ± 8.7 µm in the CM group, 30.6 ± 5.6 µm in the NTU 101 group, and 58.7 ± 16.3 µm in the LGG group. The epidermal thickness in the NOR, NTU 101, and CM groups was significantly lower than in the OVA group (*p* < 0.01) (Figure 4B). Regarding dermal layer thickness, measurements at 100× magnification revealed that the average thickness was 126.9 ± 28.8 µm in the NOR group, 172.1 ± 46.2 µm in the OVA group, 109.5 ± 44.3 µm in the CM group, 132.0 ± 21.0 µm in the NTU 101 group, and 192.8 ± 48.4 µm in the LGG group. Dermal thickness in the OVA group was significantly higher than in the NOR group (*p* < 0.05), while the NTU 101 and CM groups had significantly lower thickness compared to the OVA group (*p* < 0.05, *p* < 0.01) (Figure 4C).

**Figure 5:**
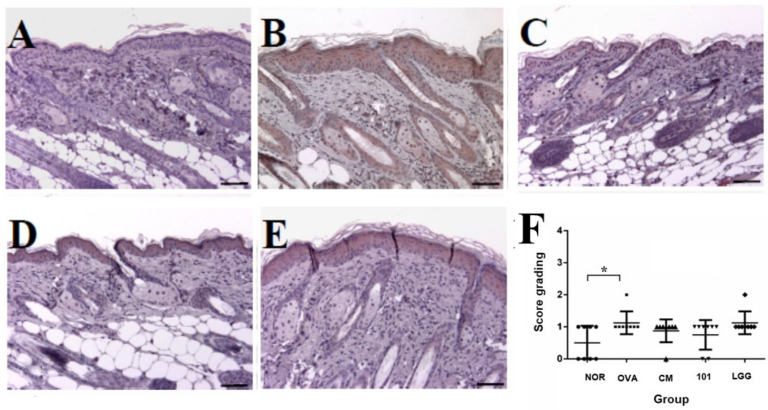
Representative images of TSLP expression in sensitized skin sites of mice. Skin sections were stained using immunohistochemistry for TSLP. Images are shown for the (**A**) NOR group, (**B**) OVA group, (**C**) CM group, (**D**) NTU 101 group, and (**E**) LGG group, and (**F**) results of scoring in each group, examined at an original magnification of 100×. Each value is expressed as mean ± SD (*n* = 8). Statistical comparison of differences between each group and the OVA group (*: *p* < 0.05). The NOR group (control) received saline and a standard diet. The OVA group underwent sensitization and skin irritation with ovalbumin (OVA) on a normal diet. The CM group had the same OVA treatment plus prednisolone. The NTU 101 group received OVA treatment and NTU 101 at 1 × 10^11^ CFU/day. The LGG group underwent OVA treatment and received *L. rhamnosus* GG ATCC 53103 at 1 × 10^11^ CFU/day.

In the analysis of TSLP antigen expression, it was observed that in the OVA-induced AD animal model, the primary site of TSLP antigen expression was the epidermal cell layer, and its expression level was correlated with the severity of AD. This experiment found that TSLP antigen expression in the OVA group was significantly higher than in the NOR group (*p* < 0.05). However, no significant differences were observed in TSLP expression levels between the treatment groups and the OVA group (Figure 5F).

### 3.4. Measurement of Total IgE and OVA-Specific IgE Antibodies in Serum

**Figure 6:**
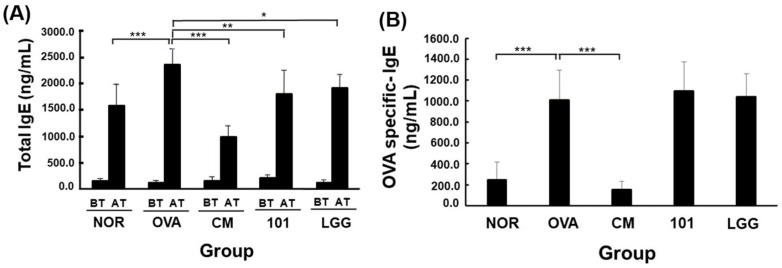
Changes in total IgE (**A**) and OVA-specific IgE (**B**) in the serum of each group. Each value was expressed as the mean ± SD (*n* = 8). Statistical comparison of differences between each group and the OVA group (*: *p* < 0.05, **: *p* < 0.01, ***: *p* < 0.001). The NOR group (control) received saline and a standard diet. The OVA group underwent sensitization and skin irritation with ovalbumin (OVA) on a normal diet. The CM group had the same OVA treatment plus prednisolone. The NTU 101 group received OVA treatment and NTU 101 at 1 × 10^11^ CFU/day. The LGG group underwent OVA treatment and received *L. rhamnosus* GG ATCC 53103 at 1 × 10^11^ CFU/day. BT: before treatment; AT: after treatment.

Serum levels of total IgE and OVA-specific IgE antibodies were measured before and after the experiment (Figure 6). Initially, total IgE levels were similar across all groups. After inducing the AD model with OVA, total IgE levels increased in all groups, with the OVA group significantly higher than the NOR group (*p* < 0.001). The NTU 101 group had significantly lower total IgE levels compared to the OVA group (*p* < 0.01) and also lower than the LGG and CM groups (*p* < 0.05; *p* < 0.001). Following OVA induction, OVA-specific IgE levels increased in the OVA, NTU 101, and LGG groups, with the OVA group significantly higher than the NOR group (*p* < 0.001). No significant differences were observed between the NTU 101, LGG, and OVA groups. However, the CM group showed a significant decrease in OVA-specific IgE levels compared to the OVA group (*p* < 0.001).

### 3.5. Animal Dissection and Tissue Observation

**Figure 7:**
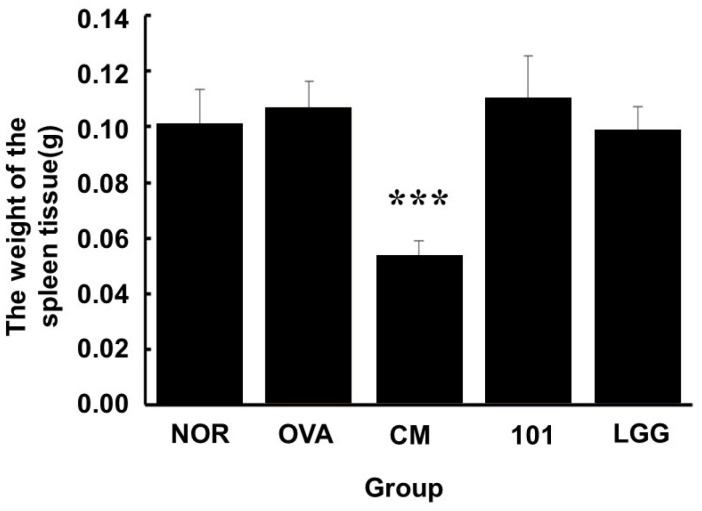
Changes in spleen tissue weight in each group at the end of the experiment. Each value is expressed as mean ± SD (*n* = 8). Statistical comparison of differences between each group: *** denotes *p*-value less than 0.001 (*p* < 0.001). The NOR group (control) received saline and a standard diet. The OVA group underwent sensitization and skin irritation with ovalbumin (OVA) on a normal diet. The CM group had the same OVA treatment plus prednisolone. The NTU 101 group received OVA treatment and NTU 101 at 1 × 10^11^ CFU/day. The LGG group underwent OVA treatment and received *L*. *rhamnosus* GG ATCC 53103 at 1 × 10^11^ CFU/day.

The results of the post-experiment dissection and observations are shown in Figure 7. During the experiment, each group of animals was continuously administered either water, NTU 101 substances, LGG powder products, or clinical medications orally. At the end of the experiment, the animals were sacrificed, and their spleen tissues were collected and weighed. The spleen weights of the animals in the OVA, NTU 101, and LGG groups were similar to those in the NOR group. However, animals in the CM group exhibited significant splenic atrophy, with spleen weights significantly lower than those in the OVA group (*p* < 0.001).

### 3.6. Cellular Subtyping and Cytokine Detection

**Figure 8:**
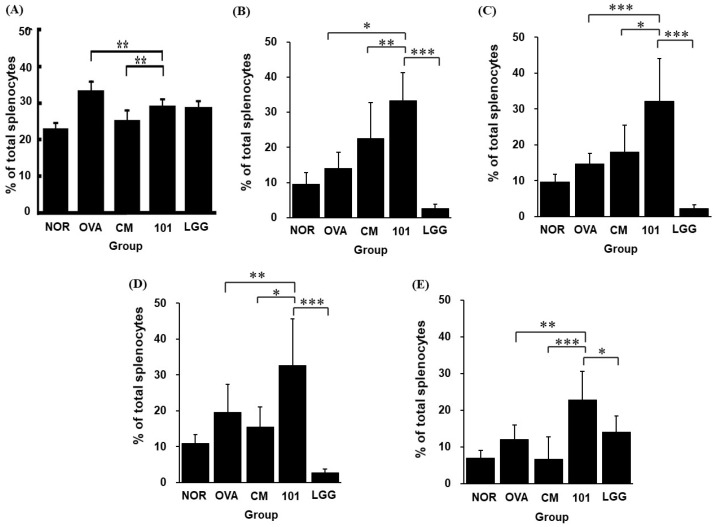
Effects of *Lactobacillus paracasei* subsp. *paracasei* NTU 101 on the cell proportions of (**A**) dead splenocytes, (**B**) CD4+/IFN-γ+, (**C**) CD4+/IL-4+, (**D**) CD4+/IL-17+, and (**E**) CD4+/CD25+/FOXP3+ in splenocytes from EC-sensitized mice. Each value is expressed as mean ± SD (*n* = 8). Statistical comparison of differences between each group: * denotes *p*-value less than 0.05 (*p* < 0.05); ** denotes *p*-value less than 0.01 (*p* < 0.01); *** denotes *p*-value less than 0.001 (*p* < 0.001). The NOR group (control) received saline and a standard diet. The OVA group underwent sensitization and skin irritation with ovalbumin (OVA) on a normal diet. The CM group had the same OVA treatment plus prednisolone. The NTU 101 group received OVA treatment and NTU 101 at 1 × 10^11^ CFU/day. The LGG group underwent OVA treatment and received *L. rhamnosus* GG ATCC 53103 at 1 × 10^11^ CFU/day.

At the end of the experiment, after sacrificing the animals, splenic tissues were harvested for cell isolation and staining. Flow cytometry was used to detect the proportions of propidium iodide (PI), CD4+/IL-4+, CD4+/IL-17+, CD4+/IFN-γ+, and CD4+/CD25+/FOXP3+ cells. Regarding the proportion of dead cells in the spleen, the OVA group exhibited the highest proportion of dead spleen cells, with all treatment groups showing significantly lower proportions compared to the OVA group (*p* < 0.01). The proportion of dead spleen cells in the NTU 101 group was significantly lower than in the OVA group (*p* < 0.01) and significantly higher than in the CM group. No significant difference was observed between the NTU 101 and LGG groups (Figure 8A).

Regarding the proportions of immune cell populations (CD4+/IL-4+, CD4+/IL-17+, CD4+/IFN-γ+, CD4+/CD25+/FOXP3+) in the spleen, the proportion of CD4+/IFN-γ+ cells (Th1 cells) in the NTU 101 group was significantly higher than in the OVA group (*p* < 0.05) and significantly higher than in the LGG and CM groups (*p* < 0.001; *p* < 0.01) (Figure 8B). The proportion of CD4+/IL-4+ cells (Th2 cells) in the NTU 101 group was significantly higher than in the OVA and LGG groups (*p* < 0.001) and higher than in the CM group (*p* < 0.05) (Figure 8C). The proportion of CD4+/IL-17+ cells (Th17 cells) in the NTU 101 group was significantly higher than in the OVA group (*p* < 0.01) and significantly higher than in the LGG and CM groups (*p* < 0.001; *p* < 0.05) (Figure 8D). Additionally, the proportion of CD4+/CD25+/FOXP3+ cells (Treg cells) in the NTU 101 group was significantly higher than in the OVA group (*p* < 0.01) and higher than in the LGG and CM groups (*p* < 0.05; *p* < 0.001) (Figure 8E).

## 4. Discussion

This study aimed to evaluate the therapeutic efficacy of NTU 101 in alleviating allergic symptoms of AD. While the pathogenesis of AD is not yet fully understood, it is believed to be influenced by environmental factors. Currently, corticosteroids are commonly used in treatment but can lead to adverse effects with long-term use. Although this study demonstrated the significant therapeutic potential of NTU 101, its long-term safety has not been fully established. Given the complexity of the gut microbiota, prolonged supplementation with a single probiotic strain could potentially disrupt the overall microbial balance. Additionally, like other probiotics, NTU 101 may carry rare but potential immune-related side effects, such as overstimulation of the immune system or triggering allergic reactions. Therefore, before advancing its clinical application, further research should focus on assessing its safety and tolerability, especially regarding long-term effects in various populations. The literature suggests that probiotics can alleviate allergy symptoms by modulating the immune system [17,19]. Furthermore, previous studies have shown that specific probiotic strains can enhance skin barrier function and reduce inflammatory responses, offering a potential alternative to conventional treatments. In this study, the effects of NTU 101 on the skin condition, histology, and immune system of AD model animals were observed through oral administration, aiming to assess its regulatory effects on AD symptoms. The findings from this research could provide valuable insights into the development of new therapeutic strategies for AD, emphasizing the role of probiotics in managing chronic inflammatory skin conditions.

Using OVA to establish an AD model in animals, the changes in food intake and body weight during the induction period were related to the treatment method. The results show that when animals undergo skin stimulation via bandaging, both food intake and body weight decrease. After the bandages were removed, food intake and body weight resumed. However, throughout the experimental period, food intake remained similar across all groups. The body weight of animals in the experimental groups was comparable to that of the normal group, with milder weight loss observed during the bandaging period. Animals in the market group exhibited lighter weight after four weeks, while animals in the clinical medication group experienced significant weight loss after drug administration, likely due to the side effects of these drugs [20]. Therefore, it is inferred that NTU 101 may help alleviate the discomfort caused by AD symptoms, leading to more stable body weight changes in the animals (Figure 2B).

Establishing an animal model of AD can be confirmed through external appearance scoring (AD SCORE) of the stimulated skin sites, histopathological analysis of skin tissue, and measurement of serum IgE antibody concentrations [16,21]. These methods provide a comprehensive assessment of AD severity and progression, ensuring the reliability of the model. The use of multiple evaluation methods also enhances the robustness of the findings, allowing for cross-validation of results. During the first skin stimulation period, the AD SCORE of the OVA group was significantly higher than that of the NOR group, confirming the successful induction of AD-like symptoms in the model. The elevated AD SCORE in the OVA group also correlates with clinical observations in human AD, further supporting the accuracy of the model in reflecting the human condition. Additionally, during the induction period, animals in the OVA group exhibited more itching, redness, and skin damage/peeling—hallmark symptoms of AD. These observations further validate the model’s accuracy in mimicking human AD symptoms. After treatment, the NTU 101 group had lower skin scores than the OVA and LGG groups and similar scores to the NOR and CM groups (Figure 3). This suggests that NTU 101 is effective in reducing AD symptoms, potentially offering a new therapeutic approach. The marked reduction in AD SCORE in the NTU 101 group highlights its potential as a beneficial intervention. Although NTU 101 has shown promising effects in improving AD symptoms and offers a potentially safer alternative to traditional corticosteroid treatments, further clinical validation is necessary. Unlike corticosteroids, the mechanisms of action and possible long-term side effects of NTU 101 are not yet fully understood. Additionally, individual responses to probiotics may vary, necessitating further investigation in larger clinical trials. Before considering NTU 101 as a treatment option, more research is needed to ensure its efficacy and safety. The comparison with the NOR and CM groups underscores the significant improvement and normalization of skin conditions in the NTU 101 group. This normalization, compared to the OVA group, provides compelling evidence of the therapeutic efficacy of NTU 101, suggesting it could be a promising candidate for further clinical development.

Histopathological results showed that, compared to the NOR group, the OVA group exhibited increased inflammatory cell infiltration and thickening of the epidermal and dermal layers. The LGG group showed slightly lower levels of inflammatory cell infiltration and skin thickening compared to the OVA group, but these levels were still higher than those in the NTU 101 group (Figure 4A–C). Skin sections from the NTU 101 group showed significantly fewer inflammatory cells, less thickening of the epidermal and dermal layers, and a trend toward lower TSLP antigen expression compared to the OVA group. The literature suggests that TSLP antigen expression positively correlates with AD severity [22]. In this experiment, the OVA group also showed increased TSLP antigen expression in the epidermal cell layer (Figure 5F). Additionally, NTU 101 treatment led to observable improvements in overall skin condition, including reduced redness, dryness, and scaling. Behavioral observations further indicated that the NTU 101 group exhibited less scratching, a common symptom of AD, compared to the OVA group. This suggests that NTU 101 not only ameliorates histopathological changes but also positively impacts the behavioral symptoms associated with AD. Overall, both observational and histopathological assessments indicate that the NTU 101 group showed greater improvement in AD symptoms than the LGG group, with effects comparable to those in the NOR and CM groups. These findings are further supported by histopathological evidence, showing that the NTU 101 group had significantly lower levels of inflammatory cell infiltration and skin thickening than the OVA group, along with a trend toward reduced TSLP antigen expression. Therefore, NTU 101 shows potential as an effective treatment for improving AD symptoms. Additionally, prednisolone, a synthetic corticosteroid widely used to treat inflammatory and autoimmune diseases, works by mimicking cortisol, a natural hormone that regulates inflammation and immune responses. While effective, prednisolone is associated with side effects [23]. In the observation of spleen tissues, the CM group exhibited splenic atrophy, likely due to the immunosuppressive effects of corticosteroids. Although this reaction is reversible, there are concerns that long-term use could impair immune function, making prolonged use in clinical settings less desirable [22].

Under normal circumstances, the immune system maintains a balance between Th1 and Th2 cells and regulates responses through regulatory T cells (Treg cells). When Th2 activity outweighs Th1 activity, allergic reactions can easily be triggered [21]. Initially, Th cells differentiate into various subtypes under the influence of different cytokines. IL-4 stimulates the differentiation of Th cells into Th2 cells, while IFN-γ, secreted by Th1 cells, inhibits Th2 cells from producing IL-4 and suppresses their differentiation. Th17 cells secrete IL-17, which induces the production of pro-inflammatory cytokines, promotes neutrophil maturation, and has chemotactic effects [24,25]. Treg cells primarily function to maintain immune tolerance and suppress excessive immune responses, thus regulating immune balance. The spleen, a secondary lymphoid organ, represents systemic immune responses. In this experiment, spleen cells were analyzed for Th cell surface markers and intracellular cytokines. In AD model animals, after the oral administration of NTU 101, the proportions of Th1, Th2, Th17, and Treg cells increased (Figure 8). Additionally, the expression of FOXP3, a specific transcription factor crucial for the maturation and immunosuppressive function of Treg cells, was also elevated [26,27]. It is inferred that NTU 101 may regulate allergic reactions associated with AD through its effects on Treg cells. Furthermore, the observed increase in Th1 cells suggests a shift toward a more balanced Th1/Th2 response, which is essential for managing allergic conditions. The enhancement of Th17 cell activity indicates an improved capacity to produce pro-inflammatory cytokines and mature neutrophils, contributing to a more effective immune response. The simultaneous increase in Treg cells and FOXP3 expression highlights NTU 101’s potential not only to suppress excessive immune reactions but also to promote immune tolerance, thereby mitigating the severity of allergic responses. This comprehensive modulation of the immune system underscores NTU 101’s therapeutic potential in treating AD and possibly other allergic diseases.

## 5. Conclusions

This study explored the mechanism of action and therapeutic efficacy of NTU 101 in an AD animal model, revealing its potential to modulate various aspects of the immune system and alleviate AD symptoms. Unlike previous studies, which primarily focused on the preventive effects of NTU 101—such as enhancing immune tolerance to avoid the onset of allergic reactions—this research shifts the focus to its therapeutic potential in treating active AD symptoms. While the preventive use of NTU 101 aims to strengthen the immune system before exposure to allergens, this study demonstrates its ability to actively modulate the immune response during the disease state. By showing that NTU 101 can increase regulatory T cells (Treg), balance the Th1/Th2 response, and enhance Th17 cell activity, this study provides new insights into NTU 101’s role in directly addressing inflammation and tissue damage, which are critical for the treatment of AD. These findings not only extend our understanding of NTU 101’s impact but also differentiate this study by focusing on its therapeutic efficacy in managing existing AD symptoms rather than solely on prevention. The multifaceted immune regulation observed in this study suggests that NTU 101 may not only serve as a standalone treatment but also as a valuable complement to conventional therapies, such as corticosteroids. Corticosteroids, while effective, are often associated with significant side effects and long-term health risks. NTU 101’s ability to enhance immune tolerance and reduce inflammation presents a potentially safer alternative or adjunctive therapy, mitigating existing symptoms while reducing the reliance on corticosteroids and their associated side effects. In addition to the findings of this study, NTU 101 has been the subject of other clinical trials that have demonstrated its positive effects on gut health and immune function in humans, providing clinical evidence supporting its safety and efficacy [28,29]. These studies further strengthen NTU 101’s potential as a therapeutic option. However, while they highlight NTU 101’s general health benefits, targeted clinical trials specifically focused on AD are essential to confirm its therapeutic value in managing this condition. In conclusion, probiotics like NTU 101 may serve as both a valuable preventive tool, by enhancing immune tolerance to allergens, and a therapeutic agent, actively treating existing AD symptoms. It may also complement corticosteroids, enhancing treatment effectiveness while reducing the side effects associated with long-term steroid use. Future research should particularly focus on identifying the risks of adverse effects and evaluating their impact on different populations, including those with compromised immune systems. These studies are crucial to confirm the viability of NTU 101 as a novel therapeutic option for AD. The findings emphasize the importance of probiotics in the management of immune-related skin disorders, paving the way for further research and development in this area.

## Data Availability

All data included in this study are available upon request by contacting the corresponding authors.
